# Mental health support within professional soccer academies: clarifying the roles of psychologists, player care staff and clinicians

**DOI:** 10.3389/fpsyg.2025.1633397

**Published:** 2025-08-01

**Authors:** Ian A. James, Martin James Turner

**Affiliations:** ^1^Burnley Football Club, Burnley, United Kingdom; ^2^School of Psychology, Manchester Metropolitan University, Manchester, United Kingdom

**Keywords:** football, mental illness, distress, psychotherapy, pathway, stepped-care

## Abstract

Surveys of professional soccer players mental health (MH) difficulties reveal a prevalence either equal or higher compared to the general population. It is suggested that under reporting of problems occurs because of the stigma associated with disclosing difficulties, poor MH literacy, and concerns about deselection. This paper presents a stepped-care pathway of MH support, together with a discussion about the optimal manner by which this support can be provided. While suggesting that enhancing the MH of players is ‘everyone’s business’, particular focus is given to the work of sports psychologists (SP), members of the player care team, and specialist MH clinicians. The use of such a pathway is discussed within a professional soccer club, and issues about communication, confidentiality and competition are debated. The pros and cons of employing a qualified MH clinician within a club are also discussed, as well as the conditions required for such an appointment to work effectively.

## Introduction

The mental health (MH) difficulties of elite athletes are receiving increasing attention, with governing bodies and professional unions developing consensus statements on the importance of monitoring and addressing athletes’ MH (Reardon et al., 2019; Gouttebarge et al., 2021). A comprehensive review of the MH difficulties of professional soccer players was undertaken by Woods et al. (2022). The reviewers identified 13 prevalence studies on MH difficulties in players. Such difficulties are often referred to in the literature as ‘common mental disorders’ (CMD). In terms of soccer, the CMD that are regularly monitored are: depression, anxiety, distress, disordered eating, sleep disturbance, substance/alcohol misuse and gambling (Gouttebarge et al., 2021). An assessment of the mental health status of professional soccer players by FIFPRO (International Federation of Professional Footballers (FIFPRO), 2025), based on studies conducted over the last 10 years, found that 38% of players experience symptoms of depression and distress; this equates to 9 soccer players in a 25-person squad. The studies also revealed that 95% of the players thought that MH difficulties negatively influenced their performances, with 65% stating their career had been affected. Over 80% of the players said there was not sufficient support during their careers to deal with their MH difficulties (International Federation of Professional Footballers (FIFPRO), 2025).

**Figure 1:**
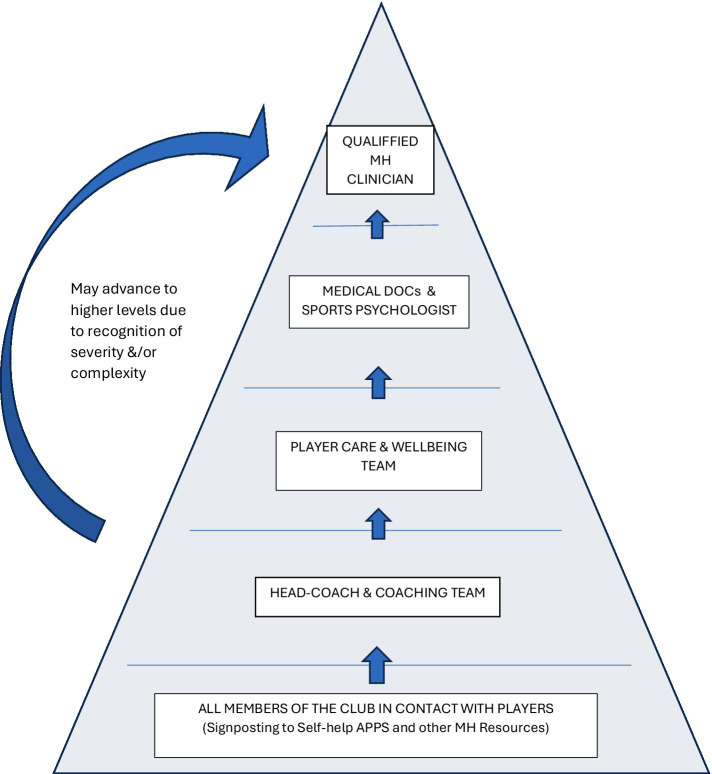
Stepped-care model of MH provision.

A previous article by the present authors provided details of the prevalence of mental distress in male and female soccer players (James et al., 2025). While the exact figures are debated (Pillay et al., 2024; Woods et al., 2022; Gouttebarge et al., 2017), it is evident that professional players experience a wide range of MH difficulties—such as: depression, anxiety, gambling, excessive drinking, disordered eating (Gouttebarge et al., 2021). The authors’ prior publication (James et al., 2025) examined the MH help-seeking behaviors of soccer players, providing a framework for looking at: Why players sought help? What help was available? Who players received help from? When was the best to time to access support? In addition, the prior article provided practical examples of how soccer clubs could support their players, using the notion that ‘MH management was everyone’s business’ from coach, physiotherapist, to psychologist. However, a specific model was also outlined which showed how the management of players’ MH difficulties could be triaged based on levels of MH severity within a multi-disciplinary team composed of sports psychologists, mental health clinicians, player care (PC) staff. Building on the previous article, the current paper proposes a stepped care referral pathway, organized as a pyramid to structure MH services within soccer clubs (Figure 1). It outlines key staff roles, the communication required between staff to enable easy movement of players between tiers, and addresses confidentiality as a critical concern (Feddersen et al., 2022). As with the first paper, the content of this article is based on a review of the literature and the authors’ personal experiences as MH providers in professional soccer clubs.

### Defining mental health

The WHO define MH as “…a state of mental well-being that enables people to cope with the stresses of life, realize their abilities, learn well and work well, and contribute to their community. It is an integral component of health and well-being […]. Mental health is more than the absence of mental disorders. It exists on a complex continuum, which is experienced differently from one person to the next […]. Mental health conditions include mental disorders and psychosocial disabilities as well as other mental states associated with significant distress.” (WHO, 2022). As noted in the definition, MH is seen as a continuum, with the majority of athletes lying between the two extremes of an ‘active mental illness and peak performance’ (Schinke et al., 2017). Steps along this continuum might include: (a) active mental illness, to (b) sub-syndromal illness (frequent symptoms), to (c) normal (occasional symptoms), to (d) good mental health (asymptomatic), and to (e) peak performance (flow or zone states) (Gulliver et al., 2012). In relation to soccer players, the latter perspective helps to recognise that players’ MH is dynamic, and it is very rarely completely negative or positive (Moore and Gardner, 2011). Keyes (2002) ‘dual’ continuum model emphasises this; with one continuum representing the absence/presence of mental illness, and the other representing degrees of mental health (Foot, 2012). From a player wellbeing perspective this would involve club staff helping players develop coping strategies to deal with mental distress, while also promoting their mental wellness. Uphill and colleagues described the latter in terms of: ‘*augmenting skills and characteristics that contribute to health and well-being, developing skills that guard against poor mental health, and building social support resources that reinforce the aforementioned* (Uphill et al., 2016).

Viewing MH as a continuum rather than a diagnostic dichotomy highlights three key areas of support that need addressing: (i) the active promotion of wellbeing, (ii) the prevention of mental illness, and (iii) the treatment of moderate to severe mental illness. Under these three headings one can see that the MH provision within a soccer club should not be limited to specialist clinicians (counsellors, clinical psychologists, psychiatrists), rather it involves a range of club staff to foster and maintain players’ mental wellbeing. The next section outlines a framework detailing who should deliver MH and wellbeing support, their roles, and the communication standards needed for the system to function effectively.

### Describing the stepped-care MH pathway

Figure 1 is a stepped-care pathway that outlines the types of support provided to players based on the severity of their mental health (MH) difficulties.

At the base of the pyramid, all performance staff—such as physiotherapists and nutritionists—can direct players to self-help resources like the FIFPRO MH website and Football Association’s MH and wellbeing materials (PFA, 2024). The next level involves coaches, who should be trained to identify low levels of distress (James et al., 2025). Kelly et al. (2018) suggest the coaches can be supported by sports psychologists (SPs) via the use of MH education programs. Above this level, the Player Care (PC) team provide assistance with player wellbeing, addressing non-sporting aspects of players’ lives, such as welfare and personal features, helping reduce off-field distractions (Austin, 2021). At the level above this, SPs and medical staff assess and support players with more complex needs. Some SPs will be trained in therapies like cognitive therapy and Acceptance and Commitment Therapy (Turner, 2022; Olusoga and Yousuf, 2023), but they have a duty of care not to work beyond their training and competencies (Dokumbilek, 2024). At the top of the pyramid is a qualified clinician (e.g., mental health nurse, counsellor, clinical psychologist, sports psychiatrist). If employed by the club, they would take responsibility and accountability for the effective delivery of the whole pathway. Their role is to assess, formulate and deliver therapies, and provide guidance and supervision to staff in the lower levels of the pathway. The stepped-care nature of the pyramid, however, suggests that most of the MH and wellbeing support will be provided by staff within the club at the lower steps of the pathway. Many ‘low level’ problems can therefore be addressed before they escalate, providing a preventative element to the model. It is important not to undervalue the potential positive MH impact of those operating at the lower end of the pyramid. Indeed, professional groups, such as physiotherapists and strength & conditioning staff, frequently form strong interpersonal bonds with players. There is recognition within the training programs of these professions that the psychosocial nature of their work, in addition to the physical features, has positive psychological impact on athletes in their care (Arvinen-Barrow et al., 2010).

Over the last few years several professional clubs in the top two tiers of the men’s and women’s game in England (Premier League, Women’s Super League, Men’s and Women’s Championships) have employed variations of the stepped-care pathway, providing players with part-time access to on-site MH clinicians. The clubs include Arsenal FC, Birmingham City FC, Brentford FC, Burnley FC, Coventry City FC, Sunderland AFC etc. Burnley’s women’s team, who currently play in the National League North, are the only team out of this list who are not currently in the highest two tiers. In contrast, most clubs in the English leagues contract external help from clinicians outside of the club to support players with severe MH difficulties. For example, a club may refer to a ‘known’ MH specialist from outside the club (counsellor, clinical psychologist, psychiatrist), or an organisation specialising in MH and sport (e.g., Sporting Chance; Cognacity). In more complex cases, players may be referred to residential clinics for a period. Each approach has advantages and drawbacks, relating to accessibility, cost, knowledge of systems, and concerns about confidentiality and disclosure (Feddersen et al., 2022), these issues are discussed in a later section.

### Reflections on the piloting of the framework

The stepped-care pyramid outlined above is currently being piloted at Burnley FC Academy (James et al., 2025), which is a Category 1 (top tier) academy as recognised by the English Premier league and Football Association (FA). Early findings from the pilot have highlighted the importance of: (i) needing to have a shared understanding amongst players, coaches, support staff about what is meant by MH and MH difficulties (ie. improving the MH literacy of players and staff), (ii) clarifying the roles and responsibilities of staff within the pyramid, (iii) fostering strong communication between departments and staff to avoid silos, and (iv) maintaining players’ confidentiality within a data-hungry sporting environment.

**Figure 2:**
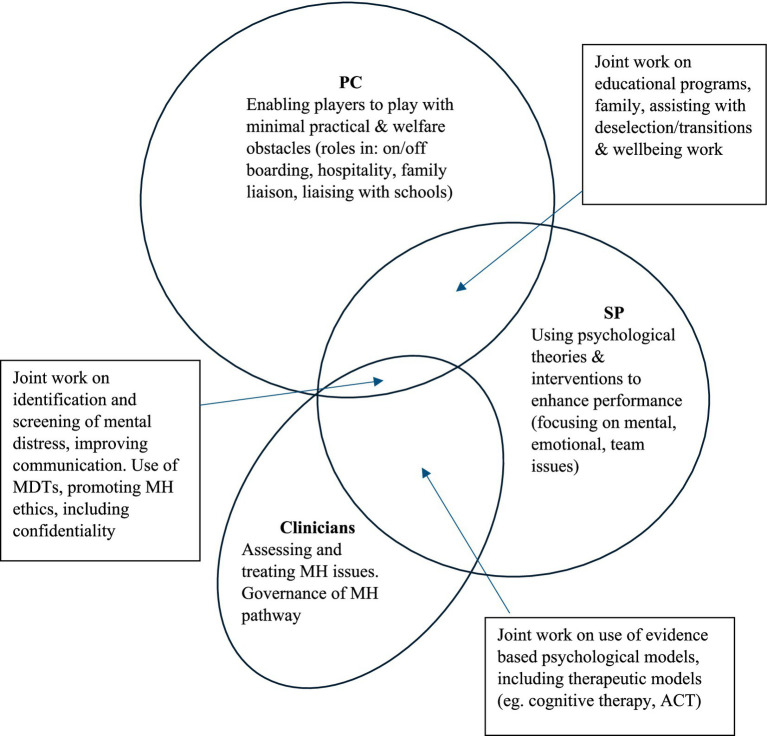
Roles of staff involved in MH provision in an academy, including joint working.

Successful implementation of the framework requires clear definition of the roles of key staff delivering wellbeing and MH services (Purcell et al., 2022). At most clubs, including Burnley FC, the main staff inputting into these areas are psychologists, members of the PC department, and MH specialists. As such it is essential that the three groups work together, recognising their commonalities and differences (O’Gorman et al., 2021). Traditionally in academies, PC staff are involved in providing practical and wellbeing support for the players, assisting with transitions, accommodation, travel arrangements, education. In an academy this may include supporting and liaising with parents/guardians, schools, health professionals, and host families of the senior players (Austin, 2021). This close contact lends itself to the development of strong relationships with players and their families, meaning that PC staff are key to the provision of pastoral care (Player Care Group, 2024). In contrast, sport psychologists (SPs) primarily deliver evidence-based interventions to enhance performance, focusing on confidence, concentration, emotional control, and communication (Harwood, 2008). Dokumbilek (2024) summarised their role as: “optimisation of athletes’ performance and enhancement of sport specific mental abilities, visualisation, goal planning and performance talk.” (page 10); a more comprehensive review of the role of SPs has been provided by Feddersen et al. (2025). While the psychologists’ and PC areas of work may appear distinct, in reality there is a great deal of overlap in roles because the performance of the players is clearly related to their wellbeing both on and off the pitch (Gallwey, 2009). This overlap between professions becomes even more pronounced when considering the contributions of MH clinicians, as captured in Figure 2. As one can see in the figure, the PC role is wide ranging, covering operational aspects, personal development and welfare features, and therefore overlaps at numerous points with the SP’s work. And all three of the professionals, evidently should work together to identify and manage players’ mental distress through collaboration and the sharing of relevant details in a confidential manner.

For the framework in Figure 1 to work effectively, strong communication between departments is essential. As outlined by James et al. (2025), there is a need for regular multidisciplinary team (MDT) meetings for sharing information and coordinating service provision. The triage model proposed in Figure 1 assigns players with complex or severe MH difficulties to clinicians, while those with milder distress are typically supported by PC staff and SPs, depending on their needs. Note, given their closer, day-to-day contact with players, PC and SP staff play a critical role in early identification and referral of those requiring specialist intervention to clinicians.

While sharing information is crucial in providing holistic, coordinated support, player confidentiality must be carefully safeguarded (Andersen et al., 2001). Stigma and shame often prevent players, especially in elite academies, from seeking help (Bu et al., 2020; Sothern and O’Gorman, 2021). Concerns about confidentiality is not merely relevant for MH issues, but also relates to the plethora of medical and performance data (scans, speed, acceleration, strength, etc) recorded and stored by clubs (Waddington and Roderick, 2002; Manley et al., 2012).

Feddersen et al. (2022) highlighted the challenges of confidentiality in a qualitative study with 16 SPs from English football academies. The findings revealed that while direct pressure to breach confidentiality was rare, indirect attempts—sometimes involving ‘trickery’—were common (Feddersen et al., 2022: Moore, 2012). Breaches also occurred when coaches, despite being explicitly asked to maintain confidentiality, shared sensitive information, such as a player’s emotional struggles after injury. To address these risks, educating all staff on the importance of confidentiality is critical to fostering a culture that encourages players to seek help (Confectioner et al., 2021).

## Discussion

Miller and colleagues’ recent review of help-seeking behaviors amongst track and field athletes recognised stigma to be the most significant barrier for athletes, because seeking help was often perceived as a sign of weakness (Miller et al., 2024; Lebrun et al., 2018). In contrast, increasing access to counsellors was found to encourage help-seeking. Due to shame and secrecy, some players will attempt to access counsellors independently of the club (James et al., 2025), which carries the risk of engaging unqualified and unregulated practitioners. To further promote help-seeking behaviors, clubs must create psychologically informed environments, offering accessible pathways run by appropriately trained and trusted staff (Castaldelli-Maia et al., 2019; Pierce et al., 2010).

In the current paper we have outlined a workable pathway, highlighting key staff involved in the MH and wellbeing services in soccer clubs (PC staff, psychologists, clinicians). It has shown areas of overlap in roles and emphasised the importance of good communication between departments. In any organisation where there is overlap, and job descriptions lack standardisation, tensions may emerge as staff navigate organisational hierarchies (Purcell et al., 2022). In the case of PC and psychology, departmental rivalries may also develop, given both disciplines are relatively new to professional football (Premier League EPPP, 2022). Notably, the FA has only recently mandated the employment of HCPC-registered psychologists in Category 1 academies, beginning with the 2024/25 season.

### Role identities of those supporting MH of players

To reduce potential tensions around MH provision, we believe it would be helpful for all departments to establish clearer ‘role identities’ (Fletcher and Wagstaff, 2009). For PC staff, this could be achieved by greater promotion of the limited number of PC training courses currently in existence. For example, one can obtain an MSc and/or a Certificate in PC from ‘Premier Sports Network/Global Institute of Sport’, or a Certificate via the ‘Player Care Group (2024). Both courses are not yet well established, and from our perspective we would encourage their curriculum to have a robust wellbeing component, covering issues such as: MH & wellbeing assessment and screening, counselling skills, ethics, and MH governance. Such content would enhance the status of the PC graduates, and provide a clearer career pathway. MH governance training would be important from a club perspective to ensure that staff protocols align with legal and ethical standards. According to Feddersen et al. (2025) recent review, SPs also need to establish a better role identify (e.g., Champ et al., 2020; Dean et al., 2022; McGlinchey et al., 2022). Feddersen et al. (2025) review of SP provision within academies calls for better integration of psychology within clubs, calling for greater organisational level input. Their conceptual guide, ‘The Sport Psychology Canvas’ identifies 10 thematic areas and asks relevant questions about the nature of psychological input in these areas. For example, Theme 5—Psychology Practice Framework—Questions: *What psychological frameworks do SP draw on (*e.g.*, counselling models)? Do SPs have any preferred intervention strategies (*e.g.*, psychological skills & educational programs)?*

The Feddersen paper does not overly focus on SPs role in MH support. Nevertheless, increasingly within the SP profession itself there is growing interest in using therapeutic approaches such as rationale emotive behavior therapy (REBT; Turner, 2022), existential therapy (Nesti, 2014), acceptance and commitment therapy (Wood and Turner, 2025). Turner et al.’s (2023) recent text on cognitive behavioral therapies is a further illustration of this trend amongst SPs.

### Role of a MH clinician

The final section of the discussion examines the role of MH clinicians within clubs. Evidently the clinicians bring expertise in terms of MH management, however, Dokumbilek (2024) suggested that they may lack the ability to apply their therapeutic models to athletes. Concerns about a lack of competence is unwarranted for some professions, for example, within psychiatry there is a special interest group called SEPSIG (Royal College of Psychiatrists Sport & Exercise Psychiatry Special Interest Group). Its members routinely work and advise professional athletes across a range of sports, including soccer (e.g., Johnston, Clinic 360). In recent years several soccer clubs have employed clinical psychologists part-time to provide therapeutic support to players and staff (Arsenal FC. Brentford FC, Burnley FC, Birmingham City FC, Coventry City FC, Sunderland AFC). Nonetheless, there are both pros and cons to employing MH clinicians within clubs; as discussed below.

In terms of pros, the advantage of hiring a clinician is that both preventative and treatment strategies can be offered (Gouttebarge et al., 2021). Clubs can clearly define the skills and qualifications required, ensuring the clinician’s expertise aligns with the needs of the coaching and sports science teams. A further advantage is that the clinician can develop relationships with stakeholders at the club, obtain a ‘helicopter’ perspective of the situation, and develop a comprehensive understanding of players’ needs. This leads to more accurate assessments, earlier identification of MH issues, and quicker access to treatment with progress monitored intimately. As outlined in Figures 1 and 2, some of the interventions can also be delegated to other members of the support team, such as SPs, PC staff, and physiotherapists. Weekly MH supervision and scaffolding can also be provided to these surrogate ‘helpers’ to ensure their MH input is in-line with the treatment goals, and joint working with the SPs can be particularly effective. The clinician can also advise on managing players during training and matches (e.g., what feedback to give/not give). Crucially, having someone on-site enables a clearer understanding of how MH difficulties affect performance, and vice versa. Keye’s (2002) dual continuum model of wellbeing (discussed previously) suggests that players with ‘well-managed’ MH issues can continue to function at high levels over periods of time. This is especially relevant in soccer, where maintaining match involvement can be critical for a player’s self-esteem. Therefore, any decision to sideline a player for MH reasons should be carefully considered.

### Problems associated with an ‘in-house’ MH clinician

Despite potential benefits, employing an ‘in-house’ clinician has some notable drawbacks. The most problematic being players’ fear that their MH disclosures may not remain confidential, potentially affecting team selection or future placement at the club (James et al., 2025). Further, the players may feel embarrassed or ashamed to be seen entering the clinician’s office, as it publicly signals to teammates and coaches that they are struggling. Players may also feel uncomfortable having to see ‘their’ therapist (who they have disclosed MH details to) on a regular basis around the training ground. Indeed, receiving therapy from an employee of the club may be seen as too big a risk for a player to take, and could ultimately prevent the above framework being used for players in the first team.

Such difficulties are less acute when a club chooses external support via a named clinician (counsellor, MH nurse, clinical psychologist, or psychiatrist) or from a specialist MH organization. The ‘external’ options provides greater perceived confidentiality because sessions are likely to be held away from club facilities. A further advantage of selecting external therapists is that the clubs are able to choose the best therapists in relation to the needs of a player. It also offers clubs the flexibility to select therapists with expertise tailored to specific player needs, such as specialists in addiction, relationship difficulties, or a clinician who can prescribe psychotropic medication (e.g., a psychiatrist).

### Embedding MH services in clubs

We believe the material above has made a cogent case for attending to the MH needs of soccer players. Gouttebarge and Kerkoffs (2020) highlight that professional clubs have a responsibility to routinely assess players’ MH, particularly during injuries and transitional periods (Woods et al., 2022). Regardless of whether clubs opt for internal or external clinicians, an effective clinical pathway requires strong endorsement from the head-coach and senior staff. Without such visible support, players are unlikely to engage with the services (Ogden et al., 2023). Crucially, this endorsement must include assurances of confidentiality to foster trust and prevent fears that disclosures will affect selection or career prospects. Lastly, Ogden et al. (2023), who studied MH difficulties in professional cricketers, advocate preventative work within academies by instilling positive MH habits and experiences from an early age. If a club decides to address the issue of MH properly, it is essential that the value of the therapeutic work is endorsed by the head-coach and their team (Ogden et al., 2023). Ideally, one would want the players to perceive working with the clinician as being beneficial to their performance, rather than an indication that they are ‘struggling mentally’.

A discussion of the MH difficulties of soccer players is incomplete without a discussion of the role of the medical department (team doctors and general practitioners). At Burnley FC Academy, medical staff are integral to the MH MDT, providing advice on physical health and pharmacological management. As outlined, Burnley FC is currently piloting the stepped-care framework, and many features of this paper reflect early experiences from that process. The MH clinician in the academy is a clinical psychologist, and it is worth noting that in addition to providing services to players the psychologist provides a clinical service to all staff throughout the club (e.g., secretaries, groundsman, departmental employees). While this broader involvement helps embed the clinician within the club culture, it also presents challenges. This is a topic for the next article in this series of MH issues in soccer clubs. Prior to concluding, it is worth examining the potential generalisability of the MH pathway (Figure 1) with respect to other soccer clubs, and across other sports. An obvious limiting factor is the cost of having a MH clinician working into an organisation. The financial feature is one of the reasons why we see MH clinicians operating in the higher tiers of the English soccer leagues. In order for the pathway to work effectively there is also a requirement to have the appropriate staff (sports psychologists, PC staff, etc.) working at the lower levels of the stepped-care model. Such a condition requires good collaboration, coordination between staff and, again, the financial resources to employ the staff highlighted in the pyramid.

## Conclusion

Mental Health (MH) provision is now seen as a requirement of all professional soccer clubs, although the manner in which it is offered varies greatly. This paper has presented a stepped-care framework that provides guidance on ‘what support should be used’ and ‘who should provide it’. The distinctive roles of Player Care (PC) staff, Sport Psychologists (SPs), and MH clinicians were outlined, alongside early insights from a pilot scheme currently underway at a professional club in England. Notwithstanding the importance of these structural and process features, the authors believe that ultimately success will depend on the establishment of a psychologically informed environment within the club. Players will only use the MH services if they trust the people and processes *in situ*. Further, the soccer players need to believe the services are beneficial to their performances and witness the MH pathway being actively endorsed by senior staff.

## Data Availability

The original contributions presented in the study are included in the article/supplementary material, further inquiries can be directed to the corresponding author.
